# Parental Smartphone Use and Children’s Emotional Well-Being in Everyday Family Life: Experience Sampling Study

**DOI:** 10.2196/95447

**Published:** 2026-10-21

**Authors:** Eva Katharina Matthias, Krisna Adiasto, Katharina Ida Salo, Lisanne S Pauw, Anne Milek

**Affiliations:** 1 Department of Psychology and Psychotherapy Faculty of Health Witten/Herdecke University Witten, North Rhine-Westphalia Germany; 2 Department of Social, Health and Organisational Psychology Faculty of Social and Behavioural Sciences Utrecht University Utrecht The Netherlands; 3 Center of Interpersonal Dynamics Witten, North Rhine-Westphalia Germany

**Keywords:** child well-being, parental phubbing, parental smartphone use, parent-child bonding, parent-child interaction, technoference

## Abstract

**Background:**

Over the past decade, smartphones have become an integral part of family life, shaping both daily routines and parent-child interactions. Although parental smartphone use in the presence of children is frequently framed as harmful through concepts such as phubbing and technoference, these constructs emphasize disruptive smartphone use and may bias interpretations toward negative effects. Moreover, most previous research has relied on cross-sectional self-report data, limiting insight into how parental smartphone use relates to children’s well-being in everyday life.

**Objective:**

This study examined the associations between parental smartphone use in the presence of the child and children’s affective well-being, as well as perceived threats to children’s fundamental needs, using both self-reported and device-logged measures of smartphone use.

**Methods:**

Individual parents (N=131) participated in a 10-day daily diary study. Each evening, parents reported their smartphone use in the presence of a selected target child, their child’s positive and negative affect, and perceived threats to their child’s fundamental psychological needs. In addition, Android users who provided consent installed a mobile sensing app that continuously recorded objective smartphone use throughout the study period. Multilevel models were used to examine both within-person (day-to-day fluctuations) and between-person associations separately for mothers and fathers while controlling for day of study, weekday vs weekend, and daily working hours.

**Results:**

Higher parental smartphone use was largely unrelated to children’s affective well-being. However, mothers who, on average, reported greater overall smartphone use perceived greater threat to their child’s fundamental needs. Similarly, on days when fathers reported using their smartphones more than usual, they also perceived greater threats to their child’s fundamental needs. In regard to positive affect, no significant associations were found for children’s positive affect perceived by their parents, indicating that parents who, on average, used their smartphones more frequently around their child did not report lower child positive affect. Regarding negative affect, fathers who reported higher overall smartphone use also reported higher child negative affect. Additionally, on days when mothers used their smartphone more than usual, they perceived their child as displaying more negative affect. None of these associations emerged when parental smartphone use was assessed using device-logged data.

**Conclusions:**

Taken together, these findings suggest that perceived child need threat may be linked to parents’ subjective perceptions and interpretations of their smartphone use rather than to their digitally traced smartphone behavior. This discrepancy underscores the importance of distinguishing between subjective and objective assessments of parental smartphone use and highlights the need for future research that integrates children’s perspectives and interactional context to clarify when and how parental smartphone use may impact children’s well-being.

## Introduction

### Overview

Over the past decade, smartphones have inevitably become part of family life, shaping both daily routines and leisure activities. While this connectivity offers many benefits, growing evidence suggests that parental smartphone use in the presence of a child may have negative consequences, as parental attention is diverted from the child to the device [1]. In the present literature, parental smartphone use is often used interchangeably with phubbing and technoference. Phubbing—a blend of “phone” and “snubbing”—refers to instances in which an individual ignores another person in a social setting by directing attention to their smartphone [2], whereas technoference refers to interruptions in social interactions resulting from smartphone use in the presence of others [3]. However, both phubbing and technoference carry a negative connotation and may, as a result of this connotation, bias interpretations, as parental smartphone use may not inherently be negative [4]. In addition, most prior studies use self-report and a cross-sectional design that only allows comparisons between people [5]. Therefore, this study leverages longitudinal self-report and mobile sensing data to obtain a more nuanced understanding of the association between parental smartphone use in the presence of children and children’s affective well-being.

### Parental Smartphone Use and Its Effects

Through the lenses of phubbing and technoference, prior work suggests that parental smartphone use in the presence of a child should have a detrimental effect on the child’s well-being [6]. Studies on phubbing indicate that phone use in the presence of a child is associated with the development of unhealthy screen habits and behaviors in the child, with especially strong implications for younger children [7,8]. These detrimental consequences may extend into children’s future lives, underscoring the importance of raising parental awareness and reducing habitual phone use in the presence of children. In line with this research, other studies found that higher levels of phubbing by one or both parents are associated with children experiencing more negative emotions and feeling disconnected from their parents [9]. Phubbing has also been linked to lower maternal happiness, which in turn predicts reduced child happiness and poorer self-regulation skills in children [10]. Meanwhile, from the perspective of technoference, higher technoference in parent-child contexts is linked to greater externalizing and internalizing behaviors [11,12], such as depressive symptoms [13] and anxiety [14], along with lower prosocial behaviors [15] and problematic smartphone use [16] among children. Furthermore, parental technoference was associated with lower self-esteem [17], higher incidence of cyberbullying [14], and higher levels of smartphone addiction [13] in adolescents. Taken together, these findings suggest that parental smartphone use in the presence of children appears to be consistently linked to poorer child well-being.

In the literature on phubbing and technoference, this detrimental effect is often explained through 2 perspectives. First, parental smartphone use in the presence of a child is thought to displace parental attention, leading to a disruption in parent-child attachment. This displacement hypothesis suggests that when parents spend more time on their smartphones, they displace the quality time they could otherwise spend with their children, which is important for fostering a secure parent-child attachment [18]. Secure attachment is often considered the hallmark of a healthy parent-child relationship [19], as it has consistently been linked to positive outcomes such as higher academic achievement, better comprehension, enhanced cognitive functioning, and stronger socioemotional competence compared to less secure attachment styles [20,21]. Consistent with the displacement hypothesis, past work suggests that children’s perceptions of parental neglect, arising from parents’ phubbing behavior, were negatively associated with the quality of parent-child attachment.

Second, the potential negative effects of parental smartphone use on children’s affective well-being can be understood through the fundamental need-threat framework proposed by Williams [22]. According to this framework, the use of a smartphone in the presence of others elicits ostracism, reflecting feelings of being invisible and/or excluded that threaten individuals’ fundamental psychological needs, thereby decreasing positive affect and increasing negative affect. Accordingly, parental smartphone use may undermine children’s affective well-being by threatening the fulfillment of children’s fundamental needs and through feelings of ostracism [23,24]. In contrast, parental engagement can fulfill children’s fundamental needs (eg, need for belonging, need for self-esteem, and sense of meaningful existence) and prevent feelings of ostracism [25-28]. Together, the displacement hypothesis and fundamental need-threat framework highlight that parental smartphone use may have tangible psychological consequences for a child, particularly when the child’s needs become unfulfilled.

A critical limitation of the above frameworks is that they are heavily derived from the phubbing and technoference literature. Both phubbing and technoference are often presented as highly negative concepts, thus biasing the overall narrative toward the idea that any parental phone use in the presence of a child will be detrimental [4]. Most measures of phubbing and technoference are negatively framed, often inquiring about moments when smartphone use was experienced as disturbing. As a result, these studies primarily capture situations in which smartphone use is indeed perceived as disruptive, which—unsurprisingly—are also associated with negative outcomes. Such framing thus fails to capture the many moments in which smartphone use is neutral or even integrated into positive interactions [5,29]. Using more neutral approaches, which do not frame parental smartphone use as phubbing or technoference, we see that the association between parental phone use and child well-being is more nuanced. On the one hand, the detrimental consequences of parental smartphone use may still be present. For instance, one study showed that child-observed parental phone use was associated with children experiencing increased anger and sadness as a result of less parental attention [30]. On the other hand, one study showed that self-reported parental smartphone use had only minimal effects on parental listening qualities, so that meaningful child-parent interactions were still possible [31]. Meanwhile, another study suggested that higher levels of social connectedness were found when parents used their phones to complement the interaction with their children [32]. Taken together, the current body of literature may thus present a skewed perspective of parental smartphone use. To obtain a more comprehensive understanding of the influence of parental smartphone use on child well-being, it may be advantageous to adopt more neutral measures that allow us to capture the full spectrum of smartphone use, including its potentially neutral or beneficial aspects.

A second limitation of prior work is its overreliance on cross-sectional data [33]. Cross-sectional studies only allow between-person comparisons, which reflect stable differences in child outcomes across parents with different levels of smartphone use. This allows us to differentiate whether higher or lower parental smartphone use is differentially linked to child well-being. However, previous research and theoretical frameworks suggest that a within-person perspective might give more insight into the processes underlying parental smartphone use. Parental smartphone use likely varies on a day-to-day basis—the same parents are likely to experience days with higher or lower smartphone use, and these fluctuations are likely to have different influences on child well-being. Consistent with this notion, one study demonstrated that there is substantial variability in daily parental smartphone use, altering the associations between parental smartphone use, technoference, and the perception of child restlessness (within-person) [5]. Another study showed no differences in parental engagement with their children between parents with higher and lower smartphone use (between-person), but higher parental smartphone use was associated with higher parental engagement at a later time (within-person) [34]. Therefore, to obtain a more comprehensive portrait of the influence of parental smartphone use on child well-being, it is beneficial to not only capture stable between-person differences but also the effects of day-to-day fluctuations in parental smartphone use.

A final limitation of past studies is that parental smartphone use has almost exclusively been measured using self-reported data [33]. Studies have shown that self-reported smartphone use data is a poor representation of actual, “objective” smartphone use data [35]. That is, findings based on self-reported amounts of smartphone use can be unreliable, as participants may misremember or underreport their behavior (eg, to appear socially acceptable). Interestingly, one of the first pioneering studies using objectively measured parental smartphone use found no association between the duration or frequency of parental smartphone use and parents’ perceptions of child restlessness [5]. Combining self-report measures with more objective markers (eg, phone logs, observer ratings, and mobile sensing) may thus yield more nuanced insights into the effects of parental smartphone use on children.

### Study Objectives

Based on these limitations, there is a recognizable need for further research that more precisely conceptualizes parental smartphone use and its interaction with children, using an objective and unbiased approach in the measurement of smartphone use [36]. Building on prior research and in response to the limitations described above, this study aims to examine the association between parental smartphone use in the presence of the child and the child’s affective well-being.

Our study makes the following contributions: first, through its intensive longitudinal, ecological momentary assessment (EMA), our study allows us to compare effects between parents and at the same time also offers insight into the (within-person) effects of day-to-day fluctuations in parental smartphone use on the child’s outcome. Second, using mobile sensing data, our study was able to compare the subjective self-report data with device-logged data. Third, by examining both parents, our study extends prior research that has predominantly focused on primary caregivers and, consequently, largely reflects the maternal perspective. Based on the above framework, this study aims to investigate the following preregistered hypotheses concerning both within-person and between-person associations between parental smartphone use in the presence of the child and children’s well-being (the preregistration is available on the Open Science Framework (OSF) [37].

At the within-person level (H1), we expected that day-to-day fluctuations in parental smartphone use would be meaningfully related to children’s affective well-being. Specifically, on days when parents used their smartphones more than usual in the presence of their child, we expected children to be rated as experiencing greater fundamental need threat, higher negative affect, and lower positive affect compared to their own average levels. This hypothesis reflects the idea that momentary increases in parental smartphone use may interfere with ongoing parent-child interactions and be associated with short-term changes in children’s affective well-being.

At the between-person level (H2), we expected that stable differences in parental smartphone use across the study period would be associated with differences in children’s overall affective well-being. That is, parents who, on average, reported higher levels of smartphone use in the presence of their child were expected to report greater fundamental need threat, higher negative affect, and lower positive affect in their children compared to parents with lower average levels of smartphone use. This hypothesis captures the assumption that more frequent exposure to parental smartphone use may be linked to more enduring differences in children’s well-being.

Finally, we additionally explored whether these associations differed between mothers and fathers, given potential differences in parenting roles, expectations, and perceptions related to smartphone use. While our preregistration referred to the construct as “parental phubbing,” we revised the terminology to “parental smartphone use” to ensure a more neutral and objective operationalization.

## Methods

### Sample

A total of 70 romantic parental couples (N=140 individuals) took part in the study. Parents were recruited via (social) media (eg, radio podcasts, a popular German psychology magazine, and social media posts), distribution of flyers in shopping malls, daycare centers, and doctoral practices, and through snowball sampling. Eligibility criteria included (1) being 18 years of age or older, (2) understanding and speaking German fluently, (3) owning a smartphone, (4) being in a romantic relationship for at least 6 months, (5) both partners agreeing to take part in the study, and (6) having a child aged 1 to 16 years. A total of 9 partners did not complete any questionnaires and were therefore not included in the analyses. This resulted in a final sample of 131 individual parents (n=61 parental couples and n=9 individual parents without a participating partner), aged between 26 and 61 years (mean 37.34, SD 6.02). The age of the target children ranged between 1 and 16 years (mean 6.59, SD 3.95, including 60/131, 46%, girls). On average, partners had been in a relationship for 10.6 (SD 6.22) years. The majority of participants were married, including 77% (50/65) of mothers and 71% (47/66) of fathers. Our compliance rate for the daily reports ranged between 83% and 86%. Additional social demographics separately for mothers and fathers are shown in Table 1.

**Table 1:** Demographic characteristics of the study sample.

Demographics			Mothers				Fathers		
			Mean (SD; min-max)		n (%)		Mean (SD; min-max)		n (%)
Parent’s age (years)			36.5 (5.41; 26-53)		65 (100)		38.1 (6.50; 27-61)		66 (100)
Target child’s age (years)			6.43 (3.87; 1-15)		65 (100)		6.74 (4.05; 1-15)		66 (100)
Number of children in the household			1.82 (0.82; 1-5)		65 (100)		1.80 (0.81; 1-5)		66 (100)
Relationship duration (years)			10.9 (6.16; 1-32)		65 (100)		10.3 (6.32; 1-32)		66 (100)
Daily working hours			2.47 (2.12; 0-7.8)		51 (78.5)		4.07 (1.93; 0-7.7)		63 (95.5)
Weekly working hours			11.1 (9.56; 0-35.1)		51 (78.5)		18.3 (8.68; 0-34.6)		63 (95.5)
Subjective socioeconomic status (1=lowest to 10=highest)			6.05 (1.53; 1-8)		65 (100)		6.26 (1.48; 2-9)		66 (100)
**Employment status**									
	Fulltime	N/A^a^		11 (17)		N/A		53 (80)	
	Halftime	N/A		38 (58)		N/A		10 (15)	
	Other (without a job, student)	N/A		16 (24)		N/A		3 (5)	
**Educational level**									
	Secondary school (Hauptschulabschluss)	N/A		3 (5)		N/A		8 (12)	
	Intermediate school (Realschulabschluss)	N/A		10 (15)		N/A		12 (18)	
	Qualification for university ([Fach-] Abitur)	N/A		20 (31)		N/A		12 (18)	
	University degree	N/A		28 (43)		N/A		28 (42)	
	PhD	N/A		4 (6)		N/A		4 (6)	

^a^N/A: not applicable.

### Procedure

Data were collected as part of the larger experience-sampling project eMotion, which combines an intensive longitudinal EMA design with mobile sensing to investigate the well-being of romantic couples in an increasingly digital world. Data collection took place between 2022 and 2025. Parents could sign up for the study online by filling out a contact form. Those who did not fulfill the inclusion criteria were automatically screened out. Those who were eligible for the study could sign up for an intake procedure, in which a trained recruiter provided information and instructions about the study. Subsequently, parents were asked to select one target child for whom the questionnaires were completed and filled out an intake questionnaire, providing informed consent and responding to demographic and trait measures. This survey took about 30-45 minutes. Couples were screened out if one or both partners did not fill out the questionnaire by midnight the day before the start of the experience sampling method (ESM) period. Then, over a period of 10 days, both parents were simultaneously beeped 5 times per day between 9:30 AM and 9:00 PM. The parents received an SMS text message with the link to the questionnaire programmed in formR [38]. An additional sixth beep included a short evening questionnaire (administered at 9:30 PM), with several (other) questions about the entire day. For this study, we only used the data from the evening questionnaire. At the end of the ESM period, parents answered a final questionnaire, including several postmeasures (ie, a few trait scales), and questions about their experience with the study. To reduce the likelihood of parents changing their target child during the study, each evening questionnaire included a reminder before the child-related questions: “When answering the following questions, please refer to the child you selected at the beginning of the study.” For the mobile sensing component, participants with Android smartphones who provided additional consent installed the movisensXS app, which continuously recorded smartphone trace data throughout the 10-day study period. At the end of the study, participants completed a final questionnaire assessing additional trait measures, were thanked and reimbursed. Furthermore, they could receive individualized feedback on their personality, burnout, emotions, emotion regulation strategy use, sleep quality and mood, partner contact and relational well-being.

### Ethical Considerations

The study procedure was approved by the ethics committee of the Psychology Department of the University of Münster, Germany, with approval number 2021-81-AM.

Written informed consent was obtained from all participants as part of the eMotion study prior to data collection. The consent covered the collection of questionnaire and smartphone sensing data and permitted the use of these data for secondary analyses. Participants were informed that participation was voluntary and that they could withdraw from the study at any time without consequences.

The data used in these analyses were deidentified prior to analysis. Participant confidentiality was maintained throughout the study, and all data were stored securely in accordance with applicable data protection regulations.

Parents received financial compensation on a pro rata basis with a maximum reimbursement of €118 (US $132.91) per person, including a €10 (US $11.26) bonus for all parents who completed more than 80% of the experience-sampling prompts. Alternatively, participating students could opt for course credit.

### Measures

#### Overview

All variables used in this project were part of the evening questionnaire and were thus answered once a day in the evening by both parents during the 10-day study period. We implemented different ways to estimate the internal consistency of our self-reported measures. For multi-item EMA measures, we first estimated Cronbach α separately for each study day. We then report the average of daily α values as a pragmatic indicator of reliability. For 2-item measures, we report mean daily interitem correlation and Spearman-Brown reliability. For single-item measures, where internal consistency is not applicable, we instead report intraclass correlation coefficient (ICC) across days.

#### Child Fundamental Need Threat

Child fundamental need threat was measured by asking participants to reflect on the extent to which their child felt “rejected” (Belonging), “it had the situation(s) under control,” “irritated” (Control), “at ease” (Self-Esteem), “invisible” (Meaningful existence), and “excluded” (Ostracism) because of their smartphone use that day. Participants rated these statements on a 7-point scale (1: “not at all”; 7: “very much”). An average of these 6 items was calculated to obtain an aggregate measure of child fundamental need threat, with higher scores reflecting higher need threat due to smartphone use. Mean daily α suggested that the internal consistency for the child fundamental need threat items was good (α=.84).

#### Child Positive Affect

Child positive affect was measured by averaging the 2 items (“My child was happy today” and “My child was relaxed today”). Before reporting on any child-related smartphone use items, parents were asked to rate these statements on a 7-point scale (1: “not at all”; 7: “very much”), with higher scores reflecting more positive affect. Mean daily interitem correlation and Spearman-Brown coefficients suggested that the internal consistency of the 2 items was acceptable (*r=*0.55; *r_SB_*=0.71).

#### Child Negative Affect

Child negative affect was assessed by averaging the following 6 items per day: “My child was sad today,” “My child was upset today,” “My child was worried today,” “My child was exhausted today,” “My child was stressed today,” and “My child was down today.” Before reporting on any child-related smartphone use items, parents rated these statements on a 7-point scale (1: “not at all”; 7: “very much”), with higher scores reflecting more negative affect. Mean daily α suggested that the internal consistency for the child negative affect items was acceptable (α=.75).

#### Self-Reported Parental Smartphone Use in the Presence of a Child

Parental smartphone use in the presence of a child was assessed using one self-report item (“To what extent did you use your smartphone today in the presence of your child? (Not counting answering the questionnaires on the smartphone)”). Both parents rated this item separately on a 7-point scale (1: “not at all”; 7: “very much”) every evening, with higher scores reflecting more parental smartphone use. The ICC for this item was relatively modest (ICC_adjusted_=0.41), suggesting that participants' patterns of phone use in the presence of their child tended to vary over time.

#### Overall Device-Logged Parental Smartphone Use

Device-logged parental smartphone use was assessed using a mobile sensing app (movisensXS [39]). The app recorded when a participant’s smartphone screen was turned “on” and when it was turned “off” throughout the entire ESM period. Similar to the procedure used in Denecker et al [5], we calculated 2 separate parental smartphone use indicators per day: “screen frequency” represents the number of times per day a participant’s phone screen was turned on. “screen duration” reflects the total amount of time (in hours) per day participants’ devices were in use. It should be noted that while parental smartphone use was captured via device-logging, we did not collect information on whether the child was present during these logged episodes. Device-logged parental smartphone use was added as an additional predictor after the completion of the preregistration. Hence, analyses including screen duration or frequency must be considered exploratory.

### Data Analysis

Our analysis plan was preregistered on OSF [37]. As preregistered, in a first step, we tested whether the same target child was chosen by both parents by matching the target child’s gender and age in both parental reports. Despite being instructed otherwise, 22% (n=29) of the parents did not choose the same target child. Hence, we refrained from using double intercept actor-partner-interdependence models to analyze our intensive longitudinal data (preregistered Strategy A). We therefore followed preregistered Strategy B, where traditional multilevel models with random intercept and random slopes for all time-varying effects would be computed separately for mothers and fathers, and for each outcome. As a result, no partner effects could be tested.

The daily evening reports constituted level 1 (L1, within-person level) and were nested within individuals at level 2 (L2, between-person level). Parental smartphone use was entered into the model as within-person (L1) and between-person (L2) components. To calculate the within-person component of parental smartphone use (L1), the variable parental smartphone use was person-mean centered. A deviation from zero can thus be interpreted as a deviation from the individual’s own average level of parental smartphone use. The between-person component (L2) was extracted by (gender-specific) grand-mean centering the within-person means of parental smartphone use. By entering this between-person component into the model, we controlled for deviations of an individual’s average level of parental smartphone use from the mean level across all mothers or fathers, respectively. The outcome variables were neither transformed nor centered. To account for potential (linear) trends of the outcome over time, we included day as a control variable, recoded such that day=0 reflected the first day of the study. We also controlled for weekday (0) or weekend day (1), to account for the possibility that parental smartphone use would differ between weekdays and weekends, since we assume that parents have more time available for their children. Additionally, hours worked in a paid job (assessed every evening) were included as control variables on level 1 (within-person centered) and level 2 (between-person centered), to account for time (not) spent with the child. Primary analyses were run using the *lme4* [40] and *lmerTest* [41] packages in R.

During the review process, the question arose whether child age might qualify our findings, as it was suggested that parental smartphone use may have more detrimental effects on younger children. Therefore, we decided to include the target child’s age as a moderator in the models. For significant interactions, we conducted simple slope analyses to further examine how the association between variables varied across different levels of children’s age. This analysis was not preregistered and should be considered exploratory. The original tables of our preregistered analysis can be found in Tables S1.1-S1.6 in Multimedia Appendix 1.

## Results

### Descriptive Statistics

Means, SDs, and zero-order correlations for all variables were calculated for the within- and between-level (separately for mothers and fathers). To test for potential differences between mothers and fathers, paired-sample t tests were conducted for all variables of interest (Tables 2 and 3). These tests revealed that mothers reported higher self-reported smartphone use in the presence of the child than fathers. Device-logged smartphone use, reflected through screen duration and screen frequency, did not significantly differ between mothers and fathers.

**Table 2:** Descriptive statistics and paired-samples t tests comparing mothers and fathers (baseline)^a^.

Variable	Mothers, mean (SD)	Fathers, mean (SD)	*t* test (*df*)	*P* value	Cohen *d*
Child fundamental need threat	3.13 (1.39)	2.63 (1.04)	1.72 (34)	.09	0.29
Child positive affect	5.01 (1.10)	5.08 (1.10)	–0.48 (51)	.63	–0.07
Child negative affect	2.15 (0.98)	2.16 (0.97)	–0.06 (51)	.96	–0.01
Self-reported smartphone use	2.70 (0.89)	2.14 (0.75)	3.92 (58)	<.001	0.51
Screen duration (hours)	4.35 (1.94)	4.20 (2.16)	0.54 (36)	.60	0.09
Screen frequency	106 (39.5)	115 (47.3)	–0.95 (36)	.35	–0.16

^a^All tests are paired-samples *t* tests. Effect sizes are reported as Cohen *d*. Missingness varied between 12% and 17%.

**Table 3:** Correlation matrix for key study between-person variables (Pearson r and 2-tailed *P* value)^a^.

Variable			Self-reported smartphone		Screen duration		Screen frequency		Fundamental need threat		Positive affect		Negative affect
**Self-reported smartphone**													
	*r*	0.11		0.06		–0.22		0.14		0.02		0.10	
	*P* value	.43		.66		.11		.36		.90		.50	
**Screen duration**													
	*r*	0.28		0.67		0.23		–0.11		–0.09		0.05	
	*P* value	.06		<.001		.09		.50		.55		.76	
**Screen frequency**													
	*r*	–0.14		0.12		0.15		0.03		0.10		0.10	
	*P* value	.35		.42		.39		.86		.49		.52	
**Fundamental need threat**													
	*r*	0.27		–0.03		0.11		0.06		–0.42		0.51	
	*P* value	.06		.87		.50		.74		.004		<.001	
**Positive affect**													
	*r*	–0.12		–0.01		–0.20		–0.23		0.58		–0.59	
	*P* value	.39		.97		.21		.10		<.001		<.001	
**Negative affect**													
	*r*	0.18		0.07		0.02		0.14		–0.58		0.51	
	*P* value	.18		.64		.88		.32		<.001		<.001	

^a^Sample sizes ranged from 31 to 60. Mothers’ correlations are below and fathers’ correlations are above the diagonal; intercorrelations between mothers and fathers are on the diagonal. The correlation is significant at the .05 level (2-tailed).

### Parental Smartphone Use and Children’s Emotional Well-Being

Below, we present the results of the different models on the association between parental smartphone use and child emotional well-being outcomes. We first present our findings for self-reported parental smartphone use, disentangling between- and within-person associations among mothers and fathers. Finally, we summarize the additional exploratory analyses linking device-logged parental smartphone use and child emotional well-being.

#### Self-Reported Parental Smartphone Use and Child Fundamental Need Threat

#### Overview

Key model estimates of the association between self-reported parental smartphone use and parental perceptions of child fundamental need threat are presented in Table 4, while a complete list of model estimates can be found in Table S2.1 in Multimedia Appendix 1.

**Table 4:** Fixed-effect estimates for within- and between-person predictors of parents’ reports of child fundamental need threat^a^.

Predictors	Need threat maternal report		Need threat paternal report	
	Estimates (95% CI)	*P* value	Estimates (95% CI)	*P* value
Self-reported smartphone use^b,c^	0.07 (–0.15 to 0.28)	.54	0.22 (0.03 to 0.42)	.03
Self-reported smartphone use^b,d^	1.13 (0.56 to 1.70)	<.001	0.46 (–0.06 to 0.99)	.08
Self-reported smartphone use^b,c^ × child age	0.02 (–0.01 to 0.05)	.15	–0.02 (–0.05 to 0.01)	.13
Self-reported smartphone use^b,d^ × child age	–0.08 (–0.15 to –0.01)	.03	–0.05 (–0.11 to 0.02)	.15
Screen duration^c^	–0.02 (–0.20 to 0.16)	.85	–0.01 (–0.13 to 0.12)	.89
Screen duration^d^	–0.01 (–0.40 to 0.39)	.98	–0.22 (–0.49 to 0.05)	.12
Screen duration^c^ × child age	0.01 (–0.02 to 0.03)	.62	0.00 (–0.02 to 0.02)	.85
Screen duration^d^ × child age	–0.01 (–0.05 to 0.03)	.73	0.02 (–0.02 to 0.06)	.25
Screen frequency^c^	–0.00 (–0.01 to 0.00)	.41	0.00 (–0.01 to 0.01)	.95
Screen frequency^d^	–0.01 (–0.03 to 0.01)	.18	–0.00 (–0.01 to 0.01)	.42
Screen frequency^c^ × child age	0.00 (–0.00 to 0.00)	.96	0.00 (–0.00 to 0.00)	.56
Screen frequency^d^ × child age	0.00 (–0.00 to 0.00)	.10	0.00 (–0.00 to 0.00)	.60

^a^In all models, time, work hours, and weekday/weekend were included as control variables.

^b^In the presence of the child.

^c^Within-person level.

^d^Between-person level.

#### Between-Person Associations

At the between-person level, self-reported parental smartphone use predicted higher levels of perceived child need threat for mothers (*b*=1.13, 95% CI 0.56-1.70; *P*<.001), but not for fathers. Crucially, the interaction between maternal smartphone use and the target children’s age was significant. Simple slope analyses indicated that higher maternal self-reported smartphone use was associated with higher perceived need threat for younger children (–1 SD; *b*=0.922, SE 0.213, 95% CI 0.496-1.349) but not for older children, although the slope remained positive (*b*=0.320, SE 0.142, 95% CI –0.042 to 0.682; Table S3.1 in Multimedia Appendix 1). Overall, this pattern of findings suggests that mothers who reported higher smartphone use tended to perceive greater need threat in their children compared to mothers with lower smartphone use, and that this association became less relevant for older children.

#### Within-Person Associations

Self-reported smartphone use was linked to child need threat for fathers (*b*=0.22, 95% CI 0.03-0.42; *P*=.03), but not for mothers. This pattern of findings thus suggests that on days when fathers reported greater smartphone use than usual, they perceived their child to display greater need threat. No statistically meaningful interaction effects of target child’s age were found for either parent.

#### Self-Reported Parental Smartphone Use and Child Positive Affect

##### Overview

Key model estimates of the association between self-reported parental smartphone use and parental perceptions of their child’s positive affect are presented in Table 5, while a complete list of model estimates can be found in Table S2.2 in Multimedia Appendix 1.

**Table 5:** Fixed-effect estimates for within- and between-person predictors of parents’ reports of child positive affect^a^.

Predictors	Positive affect maternal report			Positive affect paternal report	
	Estimates (95% CI)	*P* value	Estimates (95% CI)		*P* value
Self-reported smartphone use ^b,c^	–0.10 (–0.27 to 0.06)	.21	–0.09 (–0.27 to 0.08)		.30
Self-reported smartphone use ^c,d^	–0.15 (–0.58 to 0.28)	.48	–0.16 (–0.67 to 0.36)		.55
Self-reported smartphone use ^b,c^ × child age	0.01 (–0.01 to 0.03)	.32	0.01 (–0.01 to 0.03)		.35
Self-reported smartphone use ^c,d^ × child age	–0.01 (–0.06 to 0.05)	.78	0.03 (–0.03 to 0.09)		.33
Screen duration^c^	–0.01 (–0.20 to 0.18)	.90	–0.04 (–0.17 to 0.08)		.53
Screen duration^d^	0.20 (–0.14 to 0.55)	.24	0.07 (–0.16 to 0.29)		.55
Screen duration^c^ × child age	0.01 (–0.02 to 0.03)	.63	0.01 (–0.01 to 0.03)		.26
Screen duration^d^ × child age	–0.02 (–0.05 to 0.01)	.21	–0.01 (–0.04 to 0.01)		.29
Screen frequency^c^	0.00 (–0.01 to 0.01)	.85	–0.00 (–0.01 to 0.01)		.84
Screen frequency^d^	0.01 (–0.01 to 0.03)	.21	–0.00 (–0.01 to 0.01)		.99
Screen frequency^c^ × child age	0.00 (–0.00 to 0.00)	.69	0.00 (–0.00 to 0.00)		.96
Screen frequency^d^ × child age	–0.00 (–0.00 to 0.00)	.13	–0.00 (–0.00 to 0.00)		.59

^a^In all models, time, work hours, and weekday/weekend were included as control variables.

^b^In the presence of the child.

^c^Within-person level.

^d^Between-person level.

##### Between-Person Associations

At the between-person level, self-reported parental smartphone use was not significantly associated with perceived child positive affect for both parents. Further, no significant interactions between self-reported smartphone use and target child age were found. Overall, this pattern of findings suggests that parental perceptions of child positive affect did not differ between mothers and fathers with differing levels of smartphone use.

##### Within-Person Associations

At the within-person level, day-to-day fluctuations in self-reported parental smartphone use were also not significantly associated with perceived child positive affect for both parents. Similarly, no significant interactions between self-reported smartphone use and target child age were found. This suggests that parental perceptions of child positive affect were not contingent on day-to-day differences in parental smartphone use.

#### Self-Reported Smartphone Use and Child Negative Affect

##### Overview

Key model estimates of the association between self-reported parental smartphone use and parental perceptions of their child’s negative affect are presented in Table 6, while a complete list of model estimates can be found in Table S2.3 in Multimedia Appendix 1.

**Table 6:** Fixed-effect estimates for within- and between-person predictors of parents’ reports of child negative affect^a^.

Predictors	Negative affect maternal report			Negative affect paternal report		
	Estimates (95% CI)	*P* value	Estimates (95% CI)		*P* value	
Self-reported smartphone use ^b,c^	0.20 (0.06 to 0.34)	.006	0.04 (–0.13 to 0.20)		.66	
Self-reported smartphone use ^c,d^	0.24 (–0.09 to 0.57)	.16	0.45 (0.06 to 0.85)		.03	
Self-reported smartphone use ^b,c^ × child age	–0.02 (–0.04 to –0.00)	.04	–0.01 (–0.03 to 0.01)		.55	
Self-reported smartphone use ^c,d^ × child age	0.00 (–0.04 to 0.04)	.96	–0.05 (–0.09 to –0.00)		.04	
Screen duration^c^	–0.00 (–0.01 to 0.00)	.63	0.00 (–0.00 to 0.01)		.81	
Screen duration^d^	–0.01 (–0.02 to 0.01)	.29	0.00 (–0.01 to 0.01)		.74	
Screen duration^c^ × child age	0.00 (–0.00 to 0.00)	.37	–0.00 (–0.00 to 0.00)		.58	
Screen duration^d^ × child age	0.00 (–0.00 to 0.00)	.17	0.00 (–0.00 to 0.00)		.72	
Screen frequency^c^	0.05 (–0.10 to 0.21)	.50	0.03 (–0.07 to 0.13)		.53	
Screen frequency^d^	–0.16 (–0.43 to 0.11)	.24	0.03 (–0.15 to 0.21)		.76	
Screen frequency^c^ × child age	–0.01 (–0.02 to 0.01)	.57	–0.01 (–0.02 to 0.01)		.45	
Screen frequency^d^ × child age	0.02 (–0.00 to 0.05)	.11	0.00 (–0.02 to 0.02)		.96	

^a^In all models, time, work hours, and weekday/weekend were included as control variables.

^b^In the presence of the child.

^c^Within-person level.

^d^Between-person level.

##### Between-Person Associations

At the between-person level, self-reported parental smartphone use was significantly associated with perceived child negative affect in fathers (*b*=0.45, 95% CI 0.06-0.85; *P*=.03), but not for mothers. In addition, the interaction between paternal self-reported smartphone use and target child age was statistically significant: simple slope analyses revealed a significant positive association between paternal self-reported smartphone use and their perceptions of negative affect among younger children (–1 SD; *b*=0.320, 95% CI 0.027-0.613), but not older children (+1 SD; *b*=–0.073, 95% CI –0.315 to 0.169; Table S3.1 in Multimedia Appendix 1).

##### Within-Person Associations

Conversely, at the within-person level, self-reported parental smartphone use was significantly associated with perceived child negative affect in mothers (*b*=0.20, 95% CI 0.06-0.35; *P*=.006), but not in fathers. In addition, the interaction between maternal smartphone use and target child age was significant, suggesting that this association was prominent for younger target children (–1 SD; *b*=0.149, SE 0.052, 95% CI 0.046-0.251), but was attenuated among older target children (+1 SD; *b*=–0.010, SE 0.054, 95% CI –0.117 to 0.096). Thus, this pattern of findings suggests that on days when mothers reported higher smartphone use than usual, they perceived more negative affect in their children, but that this association became less prominent when target children were older.

#### Self-Reported vs Device-Logged Smartphone Use

We additionally investigated whether the above associations between self-reported parental smartphone use and child emotional well-being outcomes could be replicated using device-based measures of parental smartphone use, namely screen time frequency and duration. At the between- and within-person levels, these analyses revealed that neither screen time frequency nor duration were significantly associated with parental perceptions of child fundamental need threat, child positive affect, or child negative affect. This pattern of nonsignificant associations was consistent for both parents, irrespective of target child age. Key model estimates of the association between device-logged smartphone use and parental perceptions of children’s emotional well-being can be found in Tables 4-6, and a complete list of model estimates is documented in Tables S2.4-S2.6 in Multimedia Appendix 1.

## Discussion

### Summary

This study examined the association between parental smartphone use in the presence of a child and the child’s emotional well-being, using a multimethod approach that combines self-reported measures and device-based log data. By examining several indicators of child emotional well-being, our findings provide partial support to the notion that daily fluctuations in parental smartphone use are meaningfully associated with children’s well-being. For mothers, our findings show that higher-than-usual self-reported smartphone use was associated with higher child negative affect. Notably, this association was evident only among mothers with younger children, suggesting that younger children may be particularly sensitive to maternal smartphone use in their presence. Meanwhile, for fathers, higher-than-usual smartphone use was associated with greater perceived child need threat, regardless of their children’s age. Next, our findings provide partial support to the idea that stable differences in parental smartphone use are associated with differences in child emotional well-being. Mothers reporting higher smartphone use reported greater perceived child need threat, compared to mothers with lower smartphone use, and this association was particularly noticeable among mothers with younger children compared to older children. On the other hand, fathers reporting higher smartphone use reported greater perceived negative affect in their children compared to fathers with lower smartphone use. Lastly, our exploratory findings suggest that the above self-report findings are not replicable using objective, device-logged smartphone use measures. Together, this pattern of findings suggests that the association between parental smartphone use and child emotional well-being is more nuanced than presented in recent studies. Specifically, our findings suggest that parents’ subjective perceptions of their own smartphone use may be more closely related to their children’s emotional well-being than their actual, objectively recorded smartphone use.

### Between vs Within-Person Effects

By distinguishing within-person (daily fluctuations from a parent’s own average) and between-person (stable differences between parents) effects, this study scrutinizes the displacement theory and technoference framework in a daily-life setting. Displacement theory posits that time spent on smartphones reduces opportunities for parent-child interaction, thereby undermining child well-being. Similarly, technoference and phubbing research view technology-related interruptions as relational stressors that disrupt attentional availability and responsiveness [12], evoking feelings of being ostracized [23,24].

Based on these frameworks, it is reasonable to expect that these mechanisms operate at the micro level: on days when parents use their smartphones more than usual in their child’s presence, children should show lower positive affect or higher negative affect. We found this to be true for mothers in terms of negative affect. Particularly, young children are perceived as having more negative affect on days when mothers use their smartphones more than usual in their presence.

This finding is in line with observational studies documenting increased child frustration or bids for attention when parental attention is diverted to smartphones [42]. However, these findings suggest that these short-term associations are neither universal nor consistent across parents. Specifically, daily increases in smartphone use were associated with greater child negative affect only for mothers, and only among mothers with younger children. One possible explanation is that mothers, on average, spend more time in direct caregiving and emotionally salient interactions with young children than fathers. Consequently, temporary reductions in maternal attentional availability may be more noticeable to young children and may therefore be reflected more strongly in maternal perceptions of children’s affective experiences. This finding is in line with experimental research showing that interruptions due to smartphone use can temporarily reduce parental responsiveness and elicit increased bids for attention from young children, reflecting children’s sensitivity to disruptions in caregiver availability [43]. Notably, Braune-Krickau et al [43] found that similar responses were observed following nondigital interruptions (ie, paper-and-pencil tasks), suggesting that the critical mechanism is not smartphone use itself but the temporary disruption of responsive parent-child interaction. This interpretation is consistent with developmental research using the still-face paradigm, which demonstrates that even brief interruptions in caregiver responsiveness evoke distress and attempts to reengage with the caregiver [44,45].

In addition to children’s affect, we examined children’s perceived fundamental need threat. Here, a different pattern emerged. On days when fathers reported higher-than-usual smartphone use in their child’s presence, they also perceived greater threats to their child’s needs (eg, belonging, meaningful existence, and control). This association was not observed for mothers and was not moderated by child age. One possible explanation is that fathers may be more likely to perceive the relational implications of their smartphone use (ie, children’s threatened fundamental needs) than immediate changes in children’s affect. While negative affect reflects observable emotional states, perceived need threat might represent a broader interpretation of the quality of the parent-child interaction. Importantly, perceived need threat was assessed immediately after smartphone use reports and explicitly framed as attributable to that use. The temporal proximity and explicit causal framing may activate self-evaluative processes, particularly in light of prevailing societal narratives that portray parental smartphone use as problematic. Under such conditions, fathers may infer need threat as a consequence of their behavior independent of observable changes in the child’s affect. In contrast, children’s positive and negative affect were assessed prior to smartphone use reports and were not explicitly framed as consequences of that use. This temporal and conceptual separation may therefore have reduced the likelihood that affect ratings were influenced by immediate reflections on fathers’ own behavior.

At the between-person level, associations emerged between mothers’ smartphone use and perceived threat to their child’s fundamental needs, and between fathers’ smartphone use and perceived child negative affect, particularly among young children. The differing patterns for mothers and fathers may reflect differences in caregiving roles and the contexts in which mothers and fathers use their smartphones. Given that mothers, on average, take over a larger share of daily caregiving [46,47], their habitual smartphone use may more frequently coincide with emotionally salient caregiving interactions. Over time, stable patterns of reduced availability may align more closely with displacement-based mechanisms, potentially shaping the relational climate of the dyad. This interpretation is consistent with research highlighting the central role of maternal interaction quality in children’s emotional adjustment [18,48]. By contrast, the association with fathers’ smartphone use was observed for children’s negative affect rather than perceived need threat. One possible explanation is that negative affect may be more readily observable than underlying threats to children’s fundamental psychological needs.

### Children’s Age as a Moderator

An important finding of this study was that several associations between parental smartphone use and child emotional well-being were evident only among younger children. These findings suggest that child age represents an important developmental context that shapes parental perceptions of children’s susceptibility to their smartphone use. This notion is consistent with previous research showing that the detrimental effects of parental smartphone use are particularly pronounced during early childhood [7,8]. One explanation is that younger children require more direct parental care, supervision, and emotional responsiveness than older children. During early childhood, parents remain children’s primary source of emotional coregulation, social interaction, and need fulfillment. Consequently, temporary reductions in parental attentional availability due to smartphone use may be perceived by parents as particularly salient and disruptive for younger children. In contrast, as children grow older, they become increasingly autonomous, develop more advanced emotion regulation skills, and rely more heavily on peers than on parents for social interaction and support. Older children may therefore be better able to compensate for temporary reductions in parental attention, rendering them less susceptible to the immediate effects of parental smartphone use. Together, these findings suggest that displacement and technoference processes do not operate uniformly across development but are likely to be strongest during developmental periods characterized by greater dependence on parental attention and responsiveness.

### Self-Report vs Device-Logged Smartphone Use

This study used 2 distinct operationalizations of smartphone behavior: contextualized self-reports of smartphone use in the child’s presence and device-logged measures of overall smartphone use independent of context. Prior work suggests that parental smartphone use in the presence of a child—often framed in terms of technoference or parental phubbing—is inherently detrimental to children’s well-being [12,30]. However, device-logged overall use was unrelated to child affect and perceived need threat. The divergent findings between self-reported and objectively device-logged smartphone use may reflect psychological appraisal processes: awareness of one’s own smartphone use—regardless of the actual device-logged use—may increase the perception of its potentially harmful consequences for the child, thus leading to more negative ratings of child well-being. Further, greater overall smartphone use (as indexed by device-logged data) does not necessarily imply more frequent interruptions of emotionally salient parent-child interactions—particularly, given that it is not differentiated between the child being present or not. Consequently, total smartphone use may be a relatively poor proxy for the interactional processes through which smartphone use is hypothesized to affect children’s well-being.

Gender differences further underscore the importance of measurement mode. Mothers reported higher smartphone use in their child’s presence than fathers, whereas no gender differences emerged in device-logged overall use. This discrepancy suggests that mothers and fathers may use their smartphones for similar total durations, yet differ in the contextual cooccurrence of smartphone use and caregiving. Because mothers typically spend more time in direct interaction with children, their smartphone use may more frequently overlap with caregiving contexts and thus be more likely to be perceived as technoference. Moreover, stronger societal norms regarding maternal attentiveness may heighten mothers’ sensitivity to perceive their smartphone use as “phubbing” behavior, contributing to stronger associations between self-reported use and perceived need threat. In line with this interpretation, parental phubbing has been linked to negative maternal emotions [10], consistent with guilt-based or norm-based self-evaluative processes.

Taken together, the findings indicate that the impact of parental smartphone use depends critically on how smartphone behavior is operationalized. Device logs provide objective indicators of overall use but lack contextual information about whether use occurred in the presence of the child. Self-reports capture relational context but also incorporate subjective interpretation and cultural meaning. Integrating both approaches is therefore essential for avoiding overgeneralized claims about the effects of parental smartphone use on children’s affective well-being. Most importantly, future research should also integrate children’s self-reports and independent observational indicators of affect to disentangle perceived from actual need threat and to more precisely assess the role of parental reporting biases. Incorporating interactional context and multi-informant data will be essential for clarifying when and for whom parental smartphone use meaningfully relates to children’s well-being.

### Limitations, Strengths, and Future Directions

Several limitations need to be considered, offering opportunities for future studies. First, the generalizability of our findings is limited by the characteristics of our sample. We included romantic parental couples in relatively stable relationships (ie, an average relationship duration of 10 years), which may not reflect the experiences of more diverse family structures or dynamics. Moreover, parents reported, on average, high levels of positive affect and low levels of negative affect in their children, indicating a generally high level of affective well-being. This restricted range likely reduced variability in key outcome measures, thereby limiting our ability to detect associations between parental smartphone use and children’s affective responses. Importantly, children’s high affective well-being—characterized by elevated positive affect and low negative affect—may function as a resilience factor, enabling them to cope more effectively with brief disruptions in parent-child interactions [49]. As a result, parental smartphone use may be less detrimental in this sample than suggested by previous research. In contrast, children growing up in more stressed family contexts or with lower baseline affective well-being may be more vulnerable to smartphone disruptions, potentially leading to stronger associations between parental smartphone use and child affect. Future studies should therefore examine whether similar patterns emerge in samples characterized by higher parental stress, greater relational strain, or lower child affective well-being.

Next, we did not directly assess whether parental smartphone use actually resulted in interruptions of parent-child interactions, nor whether smartphone use had immediate effects on children’s positive or negative affect. Only perceived need threat was smartphone use explicitly assessed in direct relation to smartphone use in the presence of the child. Moreover, the device-logged measures reflect overall daily smartphone use rather than smartphone use specifically during interactions with the child. While higher overall usage increases the likelihood that smartphone use occurred in the child’s presence, this approach introduces substantial measurement error and does not capture the quality, timing, or interactional context of smartphone use. This distinction is particularly important given that secondary distractions are a normative part of everyday parenting. Although parents are often judged for using their smartphones as a distraction, such use may be comparable to other routine interruptions, such as attending to household chores. As Knitter and Zemp [29] noted, it may therefore be critical to differentiate between parental smartphone use that occurs during quality parent-child interactions and smartphone use during more arbitrary moments. Without this differentiation, overall measures of smartphone use may conflate potentially disruptive “phubbing” with benign or unavoidable everyday distractions. Similarly, different smartphone activities are likely to vary in the extent to which they interfere with parent-child interaction. For example, passively scrolling through social media may reduce opportunities for responsive engagement more than engaging in a video call with family members in which the child can actively participate. Consequently, the absence of associations between device-logged smartphone use and children’s well-being should not be interpreted as evidence that digitally traced smartphone use is unrelated to child well-being. Rather, it highlights the need for more context-sensitive assessments that distinguish between smartphone use occurring outside the child’s presence, smartphone use during everyday shared activities, and smartphone use that disrupts emotionally salient parent-child interactions. Future research should therefore distinguish between different smartphone activities and their interactional context to better understand which forms of parental smartphone use are most relevant for children’s emotional well-being. In addition, future studies could combine device-based smartphone logs with proximity-sensing technologies (eg, Bluetooth beacons), observational methods, or EMAs to capture not only the timing and duration of smartphone use but also its interactional context and its immediate implications for children’s lived experiences.

Another limitation refers to our use of parental report instead of self-report. We acknowledge that children’s self-reports are generally considered the preferred source for assessing subjective emotional well-being whenever developmentally appropriate [50]. However, parent-proxy reports are widely used in developmental and child psychology research, because parents are often the primary informants of children’s emotional and behavioral functioning [50]. Therefore, our findings should be interpreted as reflecting parents’ perceptions of their children’s emotional well-being rather than children’s subjective experiences. Consequently, we do not know how children themselves experienced their parents’ smartphone use, as all measures of child well-being relied on parental reports. Future research should incorporate children’s self-reported experiences to examine whether parental judgments accurately reflect children’s subjective perceptions. To more precisely assess when and how parental smartphone use affects children, future studies could combine device-based smartphone logs with proximity-sensing technologies (eg, Bluetooth beacons), observational methods, or EMAs, allowing for a more fine-grained analysis of interactional context and children’s lived experiences.

Another limitation of our study concerned the interpretation of the device-logged smartphone data. Although device logs provide an objective estimate of smartphone use and are not subject to recall bias, they do not provide information about the context in which smartphone use occurred, nor do they provide information on the reason for the smartphone use. The absence of associations between device-logged smartphone use and child well-being should therefore not be interpreted as evidence that objectively measured smartphone use is unrelated to children’s well-being. Instead, this may indicate the need for more nuanced objective measures to distinguish between smartphone use that occurs outside the child’s presence and smartphone use that takes place together with the child and is therefore not necessarily perceived as negative by the child.

A further limitation is that we relied on parents’ adherence to the target-child instruction throughout the EMA period. Although reminders were provided before child-related questions, we were unable to independently verify whether parents consistently reported on the same child across all assessments. Therefore, some uncertainty remains regarding whether target-child switching may have influenced the results.

Finally, we did not assess parental characteristics, including neuroticism and psychological distress that may influence parental reports [51]. As these parental characteristics were not measured, we cannot determine the extent to which they influenced the observed associations. Future studies would benefit from including measures of parental psychological functioning alongside child self-reports of emotional well-being to provide a more comprehensive assessment.

Despite these limitations, this study contributes to the growing research on parental smartphone use around children by incorporating several key strengths. First, we included both mothers and fathers, addressing the persistent underrepresentation of fathers in prior research on parental smartphone use. This inclusion is particularly important given that our findings revealed differential associations for mothers and fathers, suggesting that parental smartphone use may play distinct roles within family dynamics and child-rearing contexts, with potentially different implications for children’s well-being. Second, by integrating daily diary self-report data with device-logged data, this study provides both ecologically valid and more objective indicators of parental smartphone use. This multimethod approach reduces common-method bias and allows for a more robust examination of smartphone use across measurement modalities. Importantly, the daily diary design enabled the distinction between within-person (day-to-day fluctuations) and between-person (stable individual differences) effects, offering a more nuanced understanding of how parental smartphone use relates to children’s emotional well-being over time. Notably, the relatively limited evidence for negative effects of parental smartphone use on children’s well-being—particularly at the between-person level—may be related to how parental smartphone use was operationalized in this study. Rather than framing smartphone use as inherently problematic through negatively connoted constructs such as phubbing or technoference, we adopted a neutral and descriptive approach that aimed to reflect the everyday digital context in which families live. This broader perspective may have captured a wider range of everyday smartphone behaviors than measures focusing exclusively on phubbing or technoference, thereby contributing to a more nuanced understanding of how parental smartphone use relates to children’s well-being. At the same time, this approach aligns with our call for a more nuanced examination of parental smartphone use by allowing for a more fine-grained assessment of how routine smartphone use naturally occurs in family life and how it relates to different dimensions of children’s well-being. Furthermore, we assessed multiple dimensions of child well-being, including positive affect, negative affect, and fundamental need threat. This comprehensive measurement approach allows for differentiation between distinct affective and psychological outcomes, highlighting that parental smartphone use may be more strongly related to certain aspects of children’s well-being than others.

Finally, previous research investigating child age as a moderator of the association between parental smartphone use and child well-being is limited [6]. While the majority of studies have been conducted with younger children [6], evidence regarding whether younger children are indeed more susceptible to the effects of parental smartphone use remains scarce. Our inclusion of a broader child age range therefore represents a strength of this study, as it enabled us to examine age-related differences in these associations. However, the number of adolescents in our sample was relatively small. Future research should include larger samples of older children and adolescents to further investigate developmental differences in the effects of parental smartphone use. Practically, our findings suggest that parental smartphone use should not be viewed as inherently detrimental. Parents should not fear that any smartphone use in the presence of their child is inherently harmful. Rather, potential negative effects may arise from specific patterns of use, such as reduced responsiveness or interference with parent-child interactions. Our findings may help reduce parental guilt by highlighting that their smartphone use is not necessarily harmful in itself, particularly when in the presence of younger children. However, potential negative effects may arise when parental smartphone use repeatedly interferes with responsive parent-child interactions.

### Conclusion

This study examined the association between parental smartphone use in the presence of the child and children’s affective well-being using both parental self-reports and device-logged data. Taken together, our findings suggest that parental smartphone use is not uniformly or inherently detrimental to children’s well-being. Stable differences between parents who, on average, use their smartphones more frequently around their children were largely unrelated to children’s affective well-being, indicating that higher habitual smartphone use does not necessarily coincide with poorer child outcomes. In fact, our results point to a more nuanced, situational pattern: momentary increases in parental smartphone use were associated with less favorable child outcomes in some cases. This suggests that the impact of parental smartphone use may depend less on overall use in the child’s presence and more on the timing and context of use within parent-child interactions, potentially shaped by psychological processes such as parental guilt. More broadly, these findings challenge prevailing narratives that portray parental smartphone use as categorically harmful. In line with calls for a more balanced and objective perspective [4,29], our results highlight the importance of moving beyond moralized framings of technology use in families. Smartphones are embedded in daily family life and serve both disruptive and facilitative functions. Understanding their role in parent-child relationships therefore requires approaches that acknowledge this complexity rather than assuming uniformly negative effects. In sum, advancing research on parental smartphone use will require a shift toward more nuanced, context-sensitive, and theoretically grounded perspectives that distinguish between habitual use and situational dynamics, and that better reflect the realities of contemporary family life.

## Data Availability

Data will be made available on request. The project is preregistered on OSF [37].

## Appendix

Multimedia Appendix 1 Complete list of data used in this study.

## Abbreviations

EMA: ecological momentary assessment
ESM: experience sampling method
ICC: intraclass correlation coefficient
OSF: Open Science Framework
