# Supplementary material for: Parental Smartphone Use and Children’s Emotional Well-Being in Everyday Family Life: Experience Sampling Study
Source: JMIR Pediatr Parent. 2026 Oct 21;9:e95447. doi: 10.2196/95447 (PMC13735165; doi:10.2196/95447)
Supplement: Supplementary file 1 [file pediatrics_v9i1e95447_app1.docx]

**Parental Smartphone Use and Children’s Emotional Well-Being in Everyday Family Life: Experience Sampling Study**

## Supplementary Material

Table of contents

[Supplementary Material	1](#_Toc235692499)

[Section 1. Supplementary Tables Initial Preregistered Analyses	2](#_Toc235692500)

[Section 2. Supplementary Tables of the Exploratory Analyses	8](#_Toc235692501)

[Section 3. Supplementary Table of Significant Slopes	14](#_Toc235692502)

### Section 1. Supplementary Tables Initial Preregistered Analyses

**Table S1.1**

| *Fixed and Random Effect Estimates for Within and Between-Person Predictors of Parents’ Reports of Child Need Threat – Self report Parental Smartphone Use* | | | | | | | |
| --- | --- | --- | --- | --- | --- | --- | --- |
| **Predictors** | **Mothers** | | | **Fathers** | | |  |
|  | **Estimates** | **95% CI** | **p** | **Estimates** | **95% CI** | **p** |  |
| Intercept | **2.58** | **[2.30 - 2.87]** | **<0.001** | **2.37** | **[2.15 - 2.59]** | **<0.001** |  |
| Time | **-0.05** | **[-0.07 - -0.02]** | **<0.001** | **-0.06** | **[-0.08 - -0.03]** | **<0.001** |  |
| Weekend (yes/no) | -0.05 | [-0.21 - 0.12] | 0.57 | -0.12 | [-0.32 - 0.09] | 0.26 |  |
| Work hours **w** | -0.00 | [-0.04 - 0.03] | 0.82 | 0.00 | [-0.02 - 0.03] | 0.80 |  |
| Work hours **b** | -0.07 | [-0.18 - 0.05] | 0.24 | 0.00 | [-0.09 - 0.09] | 0.96 |  |
| Self-reported Smartphone Use **w** | **0.20** | **[0.09 - 0.31]** | **<0.001** | **0.10** | **[0.00 - 0.20]** | **0.05** |  |
| Self-reported Smartphone Use **b** | **0.58** | **[0.30 - 0.86]** | **<0.001** | 0.18 | [-0.08 - 0.43] | 0.17 |  |
| **Random effects** | | | | | | |  |
| σ^2^ | 0.48 | | | 0.43 | | |  |
| τ_00_ | 0.96 _id_person.a_ | | | 0.36 _id_person.a_ | | |  |
| τ_11_ | 0.07 _id_person.a.ephubc.a_wc_ | | | 0.01 _id_person.a.ephubc.a_wc_ | | |  |
| ρ_01_ | -0.51 _id_person.a_ | | | 0.97 _id_person.a_ | | |  |
| ICC | 0.67 | | | 0.49 | | |  |
| N | 62 _id_person.a_ | | | 63 _id_person.a_ | | |  |
| Observations | 458 | | | 353 | | |  |
| Marginal R^2^ / Conditional R^2^ | 0.166 / 0.721 | | | 0.057 / 0.523 | | |  |
|  | | | | | | |  |

**Table S1.2**

| *Fixed and Random Effect Estimates for Within and Between-Person Predictors of Parents’ Reports of Child Need Threat – Mobile Sensing Data* | | | | | | | | | | | | |
| --- | --- | --- | --- | --- | --- | --- | --- | --- | --- | --- | --- | --- |
| **Predictors** | **Screen Duration** | | | | | | **Screen Frequency** | | | | | |
|  | **Mothers** | | | **Fathers** | | | **Mothers** | | | **Fathers** | | |
|  | **Estimates** | **95% CI** | **p** | **Estimates** | **95% CI** | **p** | **Estimates** | **95% CI** | **p** | **Estimates** | **95% CI** | **p** |
| Intercept | **2.52** | **[2.22 - 2.83]** | **<0.001** | **2.51** | **[2.25 - 2.77]** | **<0.001** | **2.54** | **[2.23 - 2.85]** | **<0.001** | **2.49** | **[2.23 - 2.76]** | **<0.001** |
| Time | **-0.05** | **[-0.08 - -0.02]** | **0.001** | **-0.07** | **[-0.11 - -0.04]** | **<0.001** | **-0.06** | **[-0.09 - -0.03]** | **<0.001** | **-0.07** | **[-0.11 - -0.04]** | **<0.001** |
| Weekend (yes/no) | -0.10 | [-0.32 - 0.11] | 0.34 | -0.12 | [-0.35 - 0.10] | 0.29 | -0.13 | [-0.35 - 0.08] | 0.23 | -0.13 | [-0.36 - 0.10] | 0.26 |
| Work hours **w** | -0.02 | [-0.06 - 0.02] | 0.34 | -0.00 | [-0.03 - 0.03] | 0.92 | -0.02 | [-0.06 - 0.02] | 0.33 | -0.00 | [-0.04 - 0.03] | 0.81 |
| Work hours **b** | -0.06 | [-0.20 - 0.09] | 0.44 | 0.01 | [-0.10 - 0.13] | 0.80 | -0.04 | [-0.18 - 0.10] | 0.57 | 0.04 | [-0.07 - 0.15] | 0.50 |
| Screen Duration/Frequency **w** | 0.02 | [-0.05 - 0.10] | 0.56 | -0.00 | [-0.06 - 0.06] | 0.98 | -0.00 | [-0.01 - 0.00] | 0.19 | 0.00 | [-0.00 - 0.00] | 0.27 |
| Screen Duration/Frequency **b** | -0.07 | [-0.21 - 0.08] | 0.36 | -0.07 | [-0.18 - 0.04] | 0.19 | 0.00 | [-0.01 - 0.01] | 0.69 | -0.00 | [-0.01 - 0.00] | 0.47 |
| **Random Effects** | | | | | | | | | | | | |
| σ^2^ | 0.54 | | | 0.47 | | | 0.54 | | | 0.47 | | |
| τ_00_ | 0.76 _id_person.a_ | | | 0.45 _id_person.a_ | | | 0.78 _id_person.a_ | | | 0.46 _id_person.a_ | | |
| τ_11_ | 0.00 _id_person.a.duration_screen_h_wc_ | | |  | | | 0.00 _id_person.a.frequency_screen_wc_ | | |  | | |
| ρ_01_ | 0.00 _id_person.a_ | | |  | | | 0.00 _id_person.a_ | | |  | | |
| ICC |  | | | 0.49 | | |  | | | 0.49 | | |
| N | 46 _id_person.a_ | | | 54 _id_person.a_ | | | 46 _id_person.a_ | | | 54 _id_person.a_ | | |
| Observations | 318 | | | 263 | | | 318 | | | 263 | | |
| Marginal R^2^ / Conditional R^2^ | 0.084 / NA | | | 0.071 / 0.526 | | | 0.063 / NA | | | 0.059 / 0.523 | | |

**Table S1.3**

| *Fixed and Random Effect Estimates for Within and Between-Person Predictors of Parents’ Reports of Child Positive Affect – Self report Parental Smartphone Use* | | | | | | | | |
| --- | --- | --- | --- | --- | --- | --- | --- | --- |
| **Predictors** | **Mothers** | | | **Fathers** | | | |  |
|  | **Estimates** | **95% CI** | **p** | **Estimates** | **95% CI** | | **p** |  |
| Intercept | **5.16** | **[4.93 - 5.39]** | **<0.001** | **5.06** | **[4.82 - 5.29]** | | **<0.001** |  |
| Time | 0.03 | [-0.00 - 0.06] | 0.06 | -0.01 | [-0.04 - 0.02] | | 0.499 |  |
| Weekend (yes/no) | 0.19 | [-0.01 - 0.38] | 0.06 | 0.03 | [-0.19 - 0.25] | | 0.78 |  |
| Work hours **w** | -0.00 | [-0.04 - 0.03] | 0.86 | -0.02 | [-0.05 - 0.01] | | 0.17 |  |
| Work hours **b** | -0.05 | [-0.13 - 0.04] | 0.31 | 0.06 | [-0.04 - 0.15] | | 0.24 |  |
| Self-reported Smartphone Use **w** | -0.03 | [-0.12 - 0.05] | 0.43 | -0.02 | [-0.11 - 0.07] | | 0.66 |  |
| Self-reported Smartphone Use **b** | -0.21 | [-0.42 - 0.01] | 0.06 | 0.07 | [-0.17 - 0.32] | | 0.55 |  |
| **Random Effects** | | | | | | | |  |
| σ^2^ | 0.87 | | | 0.90 | |  |  |  |
| τ_00_ | 0.43 _id_person.a_ | | | 0.38 _id_person.a_ | |  |  |  |
| ICC | 0.33 | | | 0.30 | |  | |  |
| N | 63 _id_person.a_ | | | 65 _id_person.a_ | |  | |  |
| Observations | 573 | | | 572 | |  | |  |
| Marginal R^2^ / Conditional R^2^ | 0.040 / 0.357 | | | 0.015 / 0.307 | |  | |  |
|  |  | | |  | |  | |  |

**Table S1.4**

| *Fixed and Random Effect Estimates for Within and Between-Person Predictors of Parents’ Reports of Child Positive Affect – Mobile Sensing Data* | | | | | | | | | | | | |
| --- | --- | --- | --- | --- | --- | --- | --- | --- | --- | --- | --- | --- |
| **Predictors** | **Screen Duration** | | | | | | **Screen Frequency** | | | | | |
|  | **Mothers** | | | **Fathers** | | | **Mothers** | | | **Fathers** | | |
|  | **Estimates** | **95% CI** | **p** | **Estimates** | **95% CI** | **p** | **Estimates** | **95% CI** | **p** | **Estimates** | **95% CI** | **p** |
| Intercept | **5.06** | **[4.76 - 5.35]** | **<0.001** | **5.02** | **[4.76 - 5.28]** | **<0.001** | **5.04** | **[4.74 - 5.34]** | **<0.001** | **5.01** | **[4.76 - 5.27]** | **<0.001** |
| Time | 0.03 | [-0.00 - 0.07] | 0.07 | -0.01 | [-0.05 - 0.02] | 0.52 | **0.04** | **[-0.00 - 0.07]** | **0.05** | -0.01 | [-0.05 - 0.02] | 0.53 |
| Weekend (yes/no) | 0.17 | [-0.08 - 0.42] | 0.17 | 0.16 | [-0.09 - 0.40] | 0.22 | 0.18 | [-0.07 - 0.43] | 0.15 | 0.15 | [-0.10 - 0.40] | 0.23 |
| Work hours **w** | 0.01 | [-0.04 - 0.05] | 0.78 | -0.01 | [-0.05 - 0.02] | 0.43 | 0.00 | [-0.04 - 0.05] | 0.88 | -0.01 | [-0.05 - 0.02] | 0.44 |
| Work hours **b** | -0.05 | [-0.18 - 0.07] | 0.41 | 0.05 | [-0.06 - 0.16] | 0.37 | -0.05 | [-0.17 - 0.07] | 0.43 | 0.07 | [-0.04 - 0.17] | 0.23 |
| Screen Duration/Frequency **w** | 0.03 | [-0.06 - 0.12] | 0.51 | 0.02 | [-0.05 - 0.09] | 0.55 | 0.00 | [-0.00 - 0.01] | 0.37 | -0.00 | [-0.00 - 0.00] | 0.77 |
| Screen Duration/Frequency **b** | -0.02 | [-0.15 - 0.11] | 0.79 | -0.04 | [-0.14 - 0.06] | 0.40 | -0.00 | [-0.01 - 0.01] | 0.87 | -0.00 | [-0.01 - 0.00] | 0.35 |
| **Random Effects** | | | | | | | | | | | | |
| σ^2^ | 0.90 | | | 0.81 | | | 0.90 | | | 0.81 | | |
| τ_00_ | 0.52 _id_person.a_ | | | 0.44 _id_person.a_ | | | 0.52 _id_person.a_ | | | 0.43 _id_person.a_ | | |
| τ_11_ |  | | |  | | | 0.00 _id_person.a.frequency_screen_wc_ | | |  | | |
| ρ_01_ |  | | |  | | | 0.00 _id_person.a_ | | |  | | |
| ICC | 0.36 | | | 0.35 | | |  | | | 0.35 | | |
| N | 46 _id_person.a_ | | | 57 _id_person.a_ | | | 46 _id_person.a_ | | | 57 _id_person.a_ | | |
| Observations | 398 | | | 429 | | | 398 | | | 429 | | |
| Marginal R^2^ / Conditional R^2^ | 0.017 / 0.375 | | | 0.024 / 0.365 | | | 0.027 / NA | | | 0.024 / 0.364 | | |

**Table S1.5**

| *Fixed and Random Effect Estimates for Within and Between-Person Predictors of Parents’ Reports of Child Negative Affect – Self report Parental Smartphone Use* | | | | | | | |
| --- | --- | --- | --- | --- | --- | --- | --- |
| **Predictors** | **Mothers** | | | **Fathers** | | |  |
|  | **Estimates** | **95% CI** | **p** | **Estimates** | **95% CI** | **p** |  |
| Intercept | **2.13** | **[1.95 - 2.32]** | **<0.001** | **2.14** | **[1.96 - 2.31]** | **<0.001** |  |
| Time | **-0.04** | **[-0.07 - -0.02]** | **<0.001** | **-0.04** | **[-0.06 - -0.02]** | **<0.001** |  |
| Weekend (yes/no) | -0.11 | [-0.27 - 0.04] | 0.16 | -0.10 | [-0.27 - 0.07] | 0.24 |  |
| Work hours **w** | -0.01 | [-0.04 - 0.02] | 0.41 | -0.01 | [-0.04 - 0.01] | 0.19 |  |
| Work hours **b** | 0.06 | [-0.01 - 0.13] | 0.07 | -0.02 | [-0.10 - 0.05] | 0.58 |  |
| Self-reported Smartphone Use **w** | 0.07 | [-0.00 - 0.14] | 0.05 | -0.01 | [-0.09 - 0.08] | 0.89 |  |
| Self-reported Smartphone Use **b** | **0.25** | **[0.08 - 0.41]** | **0.004** | 0.08 | [-0.12 - 0.27] | 0.45 |  |
| **Random Effects** | | | | | | |  |
| σ^2^ | 0.57 | | | 0.48 | | |  |
| τ_00_ | 0.25 _id_person.a_ | | | 0.26 _id_person.a_ | | |  |
| τ_11_ | 0.01 _id_person.a.ephubc.a_wc_ | | | 0.02 _id_person.a.ephubc.a_wc_ | | |  |
| ρ_01_ | 0.04 _id_person.a_ | | | -0.01 _id_person.a_ | | |  |
| ICC | 0.31 | | | 0.37 | | |  |
| N | 63 _id_person.a_ | | | 65 _id_person.a_ | | |  |
| Observations | 573 | | | 572 | | |  |
| Marginal R^2^ / Conditional R^2^ | 0.090 / 0.373 | | | 0.029 / 0.389 | | |  |
|  |  | | |  | | |  |

**Table S1.6**

| *Fixed and Random Effect Estimates for Within and Between-Person Predictors of Parents’ Reports of Child Negative Affect – Mobile Sensing Data* | | | | | | | | | | | | |
| --- | --- | --- | --- | --- | --- | --- | --- | --- | --- | --- | --- | --- |
| **Predictors** | **Screen Duration** | | | | | | **Screen Frequency** | | | | | |
|  | **Mothers** | | | **Fathers** | | | **Mothers** | | | **Fathers** | | |
|  | **Estimates** | **95% CI** | **p** | **Estimates** | **95% CI** | **p** | **Estimates** | **95% CI** | **p** | **Estimates** | **95% CI** | **p** |
| Intercept | **2.23** | **[1.99 - 2.46]** | **<0.001** | **2.16** | **[1.96 - 2.36]** | **<0.001** | **2.23** | **[1.99 - 2.47]** | **<0.001** | **2.17** | **[1.98 - 2.37]** | **<0.001** |
| Time | **-0.06** | **[-0.09 - -0.03]** | **<0.001** | **-0.05** | **[-0.07 - -0.02]** | **<0.001** | **-0.06** | **[-0.09 - -0.03]** | **<0.001** | **-0.05** | **[-0.07 - -0.02]** | **<0.001** |
| Weekend (yes/no) | -0.08 | [-0.28 - 0.12] | 0.43 | -0.14 | [-0.33 - 0.05] | 0.14 | -0.08 | [-0.28 - 0.12] | 0.44 | -0.14 | [-0.33 - 0.05] | 0.14 |
| Work hours **w** | -0.03 | [-0.06 - 0.01] | 0.12 | -0.01 | [-0.03 - 0.02] | 0.58 | -0.03 | [-0.07 - 0.01] | 0.01 | -0.01 | [-0.03 - 0.02] | 0.62 |
| Work hours **b** | 0.06 | [-0.04 - 0.16] | 0.26 | 0.01 | [-0.07 - 0.10] | 0.75 | 0.04 | [-0.05 - 0.14] | 0.37 | -0.00 | [-0.09 - 0.08] | 0.98 |
| Screen Duration/Frequency **w** | 0.01 | [-0.06 - 0.09] | 0.70 | 0.00 | [-0.05 - 0.05] | 0.996 | 0.00 | [-0.00 - 0.00] | 0.67 | -0.00 | [-0.00 - 0.00] | 0.65 |
| Screen Duration/Frequency **b** | 0.06 | [-0.05 - 0.16] | 0.28 | 0.03 | [-0.05 - 0.11] | 0.39 | 0.00 | [-0.00 - 0.01] | 0.80 | 0.00 | [-0.00 - 0.01] | 0.19 |
| **Random Effects** | | | | | | | | | | | | |
| σ^2^ | 0.60 | | | 0.47 | | | 0.60 | | | 0.47 | | |
| τ_00_ | 0.33 _id_person.a_ | | | 0.30 _id_person.a_ | | | 0.34 _id_person.a_ | | | 0.29 _id_person.a_ | | |
| τ_11_ |  | | | 0.00 _id_person.a.duration_screen_h_wc_ | | | 0.00 _id_person.a.frequency_screen_wc_ | | |  | | |
| ρ_01_ |  | | | 0.00 _id_person.a_ | | | 0.00 _id_person.a_ | | |  | | |
| ICC | 0.36 | | |  | | |  | | | 0.38 | | |
| N | 46 _id_person.a_ | | | 57 _id_person.a_ | | | 46 _id_person.a_ | | | 57 _id_person.a_ | | |
| Observations | 398 | | | 429 | | | 398 | | | 429 | | |
| Marginal R^2^ / Conditional R^2^ | 0.056 / 0.392 | | | 0.054 / NA | | | 0.070 / NA | | | 0.041 / 0.410 | | |

### Section 2. Supplementary Tables of the Exploratory Analyses

**Table S2.1**

| *Fixed and Random Effect Estimates for Within and Between-Person Predictors of Parents’ Reports of Child Need Threat – Self report Parental Smartphone Use* | | | | | | | |
| --- | --- | --- | --- | --- | --- | --- | --- |
| **Predictors** | **Mothers** | | | **Fathers** | | |  |
|  | **Estimates** | **95% CI** | **p** | **Estimates** | **95% CI** | **p** |  |
| Intercept | **2.75** | **[2.24 – 3.26]** | **<0.001** | **2.30** | **[1.90 – 2.71]** | **<0.001** |  |
| Time | **-0.05** | **[-0.07 – -0.02]** | **<0.001** | **-0.06** | **[-0.08 – -0.03]** | **<0.001** |  |
| Weekend (yes/no) | -0.06 | [-0.23 – 0.10] | 0.45 | -0.13 | [-0.33 – 0.08] | 0.22 |  |
| Work hours **w** | -0.00 | [-0.04 – 0.03] | 0.81 | 0.00 | [-0.03 – 0.03] | 0.88 |  |
| Work hours **b** | -0.07 | [-0.18 – 0.05] | 0.25 | -0.00 | [-0.10 – 0.10] | 0.99 |  |
| Child age | -0.03 | [-0.09 – 0.04] | 0.42 | 0.01 | [-0.04 – 0.06] | 0.61 |  |
| Self-reported Smartphone Use **w** | 0.07 | [-0.15 – 0.28] | 0.54 | **0.22** | [**0.03 – 0.42**] | **0.03** |  |
| Self-reported Smartphone Use **b** | **1.13** | [**0.56 – 1.70**] | **<0.001** | 0.46 | [-0.06 – 0.99] | 0.08 |  |
| Child age * Self-reported Smartphone Use **w** | 0.02 | [-0.01 – 0.05] | 0.15 | -0.02 | [-0.05 – 0.01] | 0.13 |  |
| Child age * Self-reported Smartphone Use **b** | **-0.08** | [**-0.15 – -0.01**] | **0.03** | -0.05 | [-0.11 – 0.02] | 0.15 |  |
| **Random Effects** | | | | | | |  |
| σ2 | 0.48 | | | 0.43 | | |  |
| τ00 | 0.90 id_person.a | | | 0.43 id_person.a | | |  |
| τ11 | 0.06 id_person.a.ephubc.a_wc | | |  | | |  |
| ρ01 | -0.49 id_person.a | | |  | | |  |
| ICC | 0.65 | | | 0.50 | | |  |
| N | 62 id_person.a | | | 63 id_person.a | | |  |
| Observations | 458 | | | 353 | | |  |
| Marginal R2 / Conditional R2 | 0.212 / 0.724 | | | 0.068 / 0.537 | | |  |
|  |  | | |  | | |  |

| *Fixed and Random Effect Estimates for Within and Between-Person Predictors of Parents’ Reports of Child Positive Affect – Self report Parental Smartphone Use* | | | | | | | | | | |
| --- | --- | --- | --- | --- | --- | --- | --- | --- | --- | --- |
| **Predictors** | **Mothers** | | | | **Fathers** | | | |  |  |
|  | **Estimates** | **95% CI** | **p** | **Estimates** | | **95% CI** | **p** |  |  |  |
| Intercept | **5.29** | **[4.89 – 5.68]** | **<0.001** | **5.16** | | **[4.77 – 5.55]** | **<0.001** |  |  |  |
| Time | 0.03 | [-0.00 – 0.06] | 0.06 | -0.01 | | [-0.04 – 0.02] | 0.49 |  |  |  |
| Weekend (yes/no) | 0.17 | [-0.03 – 0.36] | 0.09 | 0.03 | | [-0.19 – 0.25] | 0.79 |  |  |  |
| Work hours **w** | -0.00 | [ -0.04 – 0.03] | 0.84 | -0.02 | | [-0.05 – 0.01] | 0.16 |  |  |  |
| Work hours **b** | -0.04 | [-0.13 – 0.05] | 0.33 | 0.05 | | [-0.04 – 0.15] | 0.29 |  |  |  |
| Child age | -0.02 | [-0.07 – 0.03] | 0.46 | -0.01 | | [-0.06 – 0.03] | 0.57 |  |  |  |
| Self-reported Smartphone Use **w** | -0.10 | [-0.27 – 0.06] | 0.21 | -0.09 | | [-0.27 – 0.08] | 0.30 |  |  |  |
| Self-reported Smartphone Use **b** | -0.15 | [-0.58 – 0.28] | 0.48 | -0.16 | | [-0.67 – 0.36] | 0.55 |  |  |  |
| Child age * Self-reported Smartphone Use **w** | 0.01 | [-0.01 – 0.03] | 0.32 | 0.01 | | [-0.01 – 0.03] | 0.35 |  |  |  |
| Child age * Self-reported Smartphone Use **b** | -0.01 | [-0.06 – 0.05] | 0.78 | 0.03 | | [-0.03 – 0.09] | 0.33 |  |  |  |
| **Random Effects** | | | | | | | | | |  |
| σ2 | 0.86 | | | | 0.90 | | | |  |  |
| τ00 | 0.44 _id_person.a_ | | | | 0.38 _id_person.a_ | | | |  |  |
| τ11 |  | | | |  | | | |  |  |
| ρ01 |  | | | |  | | | |  |  |
| ICC | 0.34 | | | | 0.30 | | | |  |  |
| N | 63 _id_person.a_ | | | | 65 _id_person.a_ | | | |  |  |
| Observations | 573 | | | | 572 | | | |  |  |
| Marginal R2 / Conditional R2 | 0.045 / 0.368 | | | | 0.025 / 0.317 | | | |  |  |
|  |  | | | |  | | | |  |  |

**Table S2.2]**

**Table S2.3**

| *Fixed and Random Effect Estimates for Within and Between-Person Predictors of Parents’ Reports of Child Negative Affect – Self report Parental Smartphone Use* | | | | | | | |
| --- | --- | --- | --- | --- | --- | --- | --- |
| **Predictors** | **Mothers** | | | **Fathers** | | |  |
|  | **Estimates** | **95% CI** | **p** | **Estimates** | **95% CI** | **p** |  |
| Intercept | **2.05** | **[1.74 – 2.35]** | **<0.001** | **2.05** | **[1.76 – 2.35]** | **<0.001** |  |
| Time | **-0.04** | **[-0.07 – -0.02]** | **<0.001** | **-0.04** | **[-0.06 – -0.02]** | **<0.001** |  |
| Weekend (yes/no) | -0.09 | [-0.24 – 0. 07] | 0.29 | -0.10 | [-0.27 – 0.07] | 0.25 |  |
| Work hours **w** | -0.01 | [-0.04 – 0.02] | 0.44 | -0.01 | [-0.04 – 0.01] | 0.19 |  |
| Work hours **b** | 0.06 | [-0.01 – 0.13] | 0.08 | -0.01 | [-0.09 – 0.06] | 0.69 |  |
| Child age | 0.01 | [-0.03 – 0.05] | 0.53 | 0.01 | [-0.03 – 0.04] | 0.59 |  |
| Self-reported Smartphone Use **w** | **0.20** | [**0.06 – 0.34**] | **0.006** | 0.04 | [-0.13 – 0.20] | 0.66 |  |
| Self-reported Smartphone Use **b** | 0.24 | [-0.09 – 0.57] | 0.16 | **0.45** | [**0.06 – 0.85**] | **0.03** |  |
| Child age * Self-reported Smartphone Use **w** | **-0.02** | [**-0.04 – -0.00**] | **0.04** | -0.01 | [-0.03 – 0.01] | 0.55 |  |
| Child age * Self-reported Smartphone Use **b** | 0.00 | [-0.04 – 0.04] | 0.96 | **-0.05** | [**-0.09 – -0.00**] | **0.04** |  |
| **Random Effects** | | | | | | |  |
| σ2 | 0.56 | | | 0.48 | | |  |
| τ00 | 0.26 _id_person.a_ | | | 0.25 _id_person.a_ | | |  |
| τ11 | 0.01 _id_person.a.ephubc.a_wc_ | | | 0.03 _id_person.a.ephubc.a_wc_ | | |  |
| ρ01 | 0.08 _id_person.a_ | | | 0.00 _id_person.a_ | | |  |
| ICC | 0.32 | | | 0.36 | | |  |
| N | 63 _id_person.a_ | | | 65 _id_person.a_ | | |  |
| Observations | 573 | | | 572 | | |  |
| Marginal R2 / Conditional R2 | 0.097 / 0.385 | | | 0.059 / 0.397 | | |  |
|  |  | | |  | | |  |

**Table S2.4**

| *Fixed and Random Effect Estimates for Within and Between-Person Predictors of Parents’ Reports of Child Need Threat – Mobile Sensing Data* | | | | | | | | | | | | |
| --- | --- | --- | --- | --- | --- | --- | --- | --- | --- | --- | --- | --- |
|  | **Screen Duration** | | | | | | **Frequency** | | | | | |
| **Predictors** | **Mothers** | | | **Fathers** | | | **Mothers** | | | **Fathers** | | |
|  | **Estimates** | **95% CI** | **p** | **Estimates** | **95% CI** | **p** | **Estimates** | **95% CI** | **p** | **Estimates** | **95% CI** | **p** |
| Intercept | 2.52 | [1.92 – 3.11] | **<0.001** | 2.38 | [1.91 – 2.85] | **<0.001** | 2.60 | [2.03 – 3.18] | **<0.001** | 2.44 | [1.97 – 2.91] | **<0.001** |
| Time | -0.05 | [-0.08 – -0.02] | **0.001** | -0.07 | [-0.11 – -0.04] | **<0.001** | -0.06 | [-0.09 – -0.03] | **<0.001** | -0.07 | [-0.11 – -0.04] | **<0.001** |
| Weekend (yes/no) | -0.11 | [-0.32 – 0.11] | 0.33 | -0.13 | [-0.35 – 0.10] | 0.28 | -0.13 | [-0.35 – 0.09] | 0.24 | -0.12 | [-0.35 – 0.11] | 0.32 |
| Work hours **w** | -0.02 | [-0.06 – 0.02] | 0.32 | -0.00 | [-0.03 – 0.03] | 0.90 | -0.02 | [-0.06 – 0.02] | 0.35 | -0.00 | [-0.04 – 0.03] | 0.89 |
| Work hours **b** | -0.06 | [-0.21 – 0.09] | 0.42 | 0.03 | [-0.09 – 0.14] | 0.66 | -0.03 | [-0.17 – 0.12] | 0.72 | 0.04 | [-0.08 – 0.15] | 0.53 |
| Child age | 0.00 | [-0.07 – 0.08] | 0.94 | 0.02 | [-0.04 – 0.08] | 0.56 | 0.00 | [-0.07 – 0.07] | 0.98 | 0.01 | [-0.05 – 0.07] | 0.76 |
| Frequency/duration screen wc | -0.02 | [-0.20 – 0.16] | 0.85 | -0.01 | [-0.13 – 0.12] | 0.89 | -0.00 | [-0.01 – 0.00] | 0.41 | 0.00 | [-0.01 – 0.01] | 0.95 |
| Frequency/duration screen bc | -0.01 | [-0.40 – 0.39] | 0.98 | -0.22 | [-0.49 – 0.05] | 0.12 | -0.01 | [-0.03 – 0.01] | 0.18 | -0.00 | [-0.01 – 0.01] | 0.42 |
| Child age * Frequency/duration screen wc | 0.01 | [-0.02 – 0.03] | 0.62 | 0.00 | [-0.02 – 0.02] | 0.85 | 0.00 | [-0.00 – 0.00] | 0.96 | 0.00 | [-0.00 – 0.00] | 0.56 |
| Child age * Frequency/duration screen bc | -0.01 | [-0.05 – 0.03] | 0.73 | 0.02 | [-0.02 – 0.06] | 0.25 | 0.00 | [-0.00 – 0.00] | 0.10 | 0.00 | [-0.00 – 0.00] | 0.60 |
| **Random effects** | | | | | | | | | | | | |
| σ^2^ | 0.54 | | | 0.47 | | | 0.54 | | | 0.47 | | |
| τ_00_ | 0.80 _id_person.a_ | | | 0.45 _id_person.a_ | | | 0.76 _id_person.a_ | | | 0.48 _id_person.a_ | | |
| τ_11_ | 0.00 _id_person.a.duration_screen_h_wc_ | | |  | | | 0.00 _id_person.a.frequency_screen_wc_ | | |  | | |
| ρ_01_ | 0.00 _id_person.a_ | | |  | | | 0.00 _id_person.a_ | | |  | | |
| ICC |  | | | 0.49 | | |  | | | 0.51 | | |
| N | 46 _id_person.a_ | | | 54 _id_person.a_ | | | 46 _id_person.a_ | | | 54 _id_person.a_ | | |
| Observations | 318 | | | 263 | | | 318 | | | 263 | | |
| Marginal R^2^ / Conditional R^2^ | 0.090 / NA | | | 0.081 / 0.531 | | | 0.140 / NA | | | 0.063 / 0.536 | | |

**Table S2.5**

| *Fixed and Random Effect Estimates for Within and Between-Person Predictors of Parents’ Reports of Child Positive Affect – Mobile Sensing Data* | | | | | | | | | | | | | | | | | | | |
| --- | --- | --- | --- | --- | --- | --- | --- | --- | --- | --- | --- | --- | --- | --- | --- | --- | --- | --- | --- |
|  | Screen Duration | | | | | | | | Frequency | | | | | | | | | | |
| Predictors | Mothers | | | Fathers | | | | | Mothers | | | | | | Fathers | | | | |
|  | Estimates | 95% CI | p | | Estimates | | 95% CI | p | | Estimates | | 95% CI | p | Estimates | | | 95% CI | p |  |
| **Intercept** | **5.20** | **[4.68 – 5.73]** | **<0.001** | | **5.10** | | **[4.66 – 5.50]** | **<0.001** | | **5.11** | | **[4.59 – 5.63]** | **<0.001** | **5.10** | | | **[4.66 – 5.54]** | **<0.001** |  |
| Time | 0.04 | [-0.00 – 0.07] | 0.06 | | -0.01 | | [-0.05 – 0.02] | 0.50 | | 0.04 | | [-0.00 – 0.07] | 0.05 | -0.01 | | | [-0.05 – 0.02] | 0.54 |  |
| Weekend (yes/no) | 0.16 | [-0.06 – 0.38] | 0.16 | | 0.16 | | [-0.09 – 0.41] | 0.20 | | 0.18 | | [-0.07 – 0.43] | 0.16 | 0.15 | | | [-0.10 – 0.40] | 0.23 |  |
| Work hours **w** |  |  |  | | -0.01 | | [-0.05 – 0.02] | 0.41 | | 0.00 | | [-0.04 – 0.05] | 0.91 | -0.01 | | | [-0.05 – 0.02] | 0.44 |  |
| Work hours **b** |  |  |  | | 0.05 | | [-0.06 – 0.16] | 0.41 | | -0.06 | | [-0.18 – 0.07] | 0.35 | 0.06 | | | [-0.05 – 0.17] | 0.25 |  |
| Child age | -0.02 | [-0.08 – 0.05] | 0.62 | | -0.01 | | [-0.06 – 0.05] | 0.73 | | -0.02 | | [-0.08 – 0.05] | 0.58 | -0.01 | | | [-0.07 – 0.04] | 0.64 |  |
| Frequency/duration screen wc | -0.01 | [-0.20 – 0.18] | 0.90 | | -0.04 | | [-0.17 – 0.08] | 0.53 | | 0.00 | | [-0.01 – 0.01] | 0.85 | -0.00 | | | [-0.01 – 0.01] | 0.84 |  |
| Frequency/duration screen bc | 0.20 | [-0.14 – 0.55] | 0.24 | | 0.07 | | [-0.16 – 0.29] | 0.55 | | 0.01 | | [-0.01 – 0.03] | 0.21 | -0.00 | | | [-0.01 – 0.01] | 0.998 |  |
| Child age * Frequency/duration screen wc | 0.01 | [-0.02 – 0.03] | 0.63 | | 0.01 | | [-0.01 – 0.03] | 0.26 | | 0.00 | | [-0.00 – 0.00] | 0.69 | 0.00 | | | [-0.00 – 0.00] | 0.96 |  |
| Child age * Frequency/duration screen bc | -0.02 | [-0.05 – 0.01] | 0.21 | | -0.01 | | [-0.04 – 0.01] | 0.29 | | -0.00 | | [-0.00 – 0.00] | 0.13 | -0.00 | | | [-0.00 – 0.00] | 0.59 |  |
| **Random effects** | | | | | | | | | | | | | | | | | | | |
| σ^2^ | 0.90 | | | | | 0.81 | | | | | 0.90 | | | | | 0.81 | | | |
| τ_00_ | 0.51 _id_person.a_ | | | | | 0.44 _id_person.a_ | | | | | 0.51 _id_person.a_ | | | | | 0.45 _id_person.a_ | | | |
| τ_11_ |  | | | | |  | | | | | 0.00 _id_person.a.frequency_screen_wc_ | | | | |  | | | |
| ρ_01_ |  | | | | |  | | | | | 0.00 _id_person.a_ | | | | |  | | | |
| ICC | 0.36 | | | | | 0.35 | | | | |  | | | | | 0.36 | | | |
| N | 46 _id_person.a_ | | | | | 57 _id_person.a_ | | | | | 46 _id_person.a_ | | | | | 57 _id_person.a_ | | | |
| Observations | 398 | | | | | 429 | | | | | 398 | | | | | 429 | | | |
| Marginal R^2^ / Conditional R^2^ | 0.025 / 0.377 | | | | | 0.035 / 0.376 | | | | | 0.065 / NA | | | | | 0.029 / 0.375 | | | |

**Table S2.6**

| *Fixed and Random Effect Estimates for Within and Between-Person Predictors of Parents’ Reports of Child Negative Affect – Mobile Sensing Data* | | | | | | | | | | | | | |
| --- | --- | --- | --- | --- | --- | --- | --- | --- | --- | --- | --- | --- | --- |
|  | **Screen Duration** | | | | | | **Frequency** | | | | | | |
| **Predictors** | **Mothers** | | | **Fathers** | | | **Mothers** | | | **Fathers** | | | |
|  | **Estimates** | **95% CI** | **p** | **Estimates** | **95% CI** | **p** | **Estimates** | **95% CI** | **p** | | **Estimates** | **95% CI** | **p** |
| Intercept | **2.06** | **[1.64 – 2.48]** | **<0.001** | **2.11** | **[1.76 – 2.46]** | **<0.001** | **2.06** | **[1.64 – 2.48]** | **<0.001** | | **2.11** | **[1.77 – 2.46]** | **<0.001** |
| Time | **-0.06** | **[-0.09 – -0.04]** | **<0.001** | **-0.05** | **[-0.07 – -0.02]** | **<0.001** | **-0.06** | **[-0.09 – -0.03]** | **<0.001** | | **-0.05** | **[-0.07 – -0.02]** | **<0.001** |
| Weekend (yes/no) | -0.08 | [-0.28 – 0.12] | 0.44 | -0.15 | [-0.34 – 0.04] | 0.13 | -0.08 | [-0.28 – 0.13] | 0.46 | | -0.14 | [-0.34 – 0.05] | 0.14 |
| Work hours **w** | -0.03 | [-0.06 – 0.01] | 0.13 | -0.01 | [-0.03 – 0.02] | 0.59 | -0.03 | [-0.07 – 0.01] | 0.09 | | -0.01 | [-0.03 – 0.02] | 0.61 |
| Work hours **b** | 0.06 | [-0.04 – 0.17] | 0.21 | 0.02 | [-0.07 – 0.10] | 0.73 | 0.05 | [-0.05 – 0.15] | 0.33 | | 0.00 | [-0.09 – 0.09] | 0.989 |
| Child age | 0.02 | [-0.03 – 0.07] | 0.47 | 0.01 | [-0.04 – 0.05] | 0.71 | 0.03 | [-0.02 – 0.08] | 0.24 | | 0.01 | [-0.03 – 0.05] | 0.66 |
| Frequency/duration screen wc | 0.05 | [-0.10 – 0.21] | 0.50 | 0.03 | [-0.07 – 0.13] | 0.53 | -0.00 | [-0.01 – 0.00] | 0.63 | | 0.00 | [-0.00 – 0.01] | 0.81 |
| Frequency/duration screen bc | -0.16 | [-0.43 – 0.11] | 0.24 | 0.03 | [-0.15 – 0.21] | 0.76 | -0.01 | [-0.02 – 0.01] | 0.29 | | 0.00 | [-0.01 – 0.01] | 0.74 |
| Child age * Frequency/duration screen wc | -0.01 | [-0.02 – 0.01] | 0.57 | -0.01 | [-0.02 – 0.01] | 0.45 | 0.00 | [-0.00 – 0.00] | 0.37 | | -0.00 | [-0.00 – 0.00] | 0.58 |
| Child age * Frequency/duration screen bc | 0.02 | [-0.00 – 0.05] | 0.11 | 0.00 | [-0.02 – 0.02] | 0.959 | 0.00 | [-0.00 – 0.00] | 0.17 | | 0.00 | [-0.00 – 0.00] | 0.72 |
| **Random effects** | | | | | | | | | | | | | |
| σ^2^ | 0.60 | | | 0.47 | | | 0.60 | | | 0.47 | | | |
| τ_00_ | 0.32 _id_person.a_ | | | 0.31 _id_person.a_ | | | 0.33 _id_person.a_ | | | 0.30 _id_person.a_ | | | |
| τ_11_ |  | | | 0.00 _id_person.a.duration_screen_h_wc_ | | |  | | |  | | | |
| ρ_01_ |  | | | 0.00 _id_person.a_ | | |  | | |  | | | |
| ICC | 0.35 | | |  | | | 0.36 | | | 0.39 | | | |
| N | 46 _id_person.a_ | | | 57 _id_person.a_ | | | 46 _id_person.a_ | | | 57 _id_person.a_ | | | |
| Observations | 398 | | | 429 | | | 398 | | | 429 | | | |
| Marginal R^2^ / Conditional R^2^ | 0.081 / 0.398 | | | 0.056 / NA | | | 0.074 / 0.403 | | | 0.043 / 0.418 | | | |

### Section 3. Supplementary Table of Significant Slopes]

**Table S3.1]**

*Simple slopes for significant interactions between parental smartphone use and child age*

| **Parent** | **Outcome** | **Level** | **Measure** | **Child age** | **b** | **SE** | **95% CI** |
| --- | --- | --- | --- | --- | --- | --- | --- |
| Mother | Need threat | Between | Self-report | **2.62** | **0.922** | **0.213** | **[0.496 - 1.349]** |
|  |  |  |  | **6.44** | **0.621** | **0.142** | **[0.336 - 0.906]** |
|  |  |  |  | 10.27 | 0.320 | 0.181 | [-0.042 - 0.682] |
| Mother | Negative Affect | Within | Self-report | **2.62** | **0.149** | **0.052** | **[0.046 - 0.251]** |
|  |  |  |  | 6.44 | 0.069 | 0.037 | [-0.003 - 0.141] |
|  |  |  |  | 10.27 | -0.010 | 0.054 | [-0.117 - 0.096] |
| Father | Negative Affect | Between | Self-report | **2.69** | **0.320** | **0.147** | **[0.027 - 0.613]** |
|  |  |  |  | 6.72 | 0.124 | 0.098 | [-0.073 - 0.320] |
|  |  |  |  | 10.74 | -0.073 | 0.121 | [-0.315 - 0.169] |
